# COVID-19-Pandemie-bedingte Belastungen und SARS-CoV-2-Prävalenz in Pflegeeinrichtungen

**DOI:** 10.1007/s00391-021-01931-6

**Published:** 2021-07-14

**Authors:** Lukas Perkhofer, Peter Grünke, Edonjeta Gashi-Ymeri, Teresa Grünke, Joris Kroschel, Detlef Michel, Elke Pensel, Andreas Rost, Michael Denkinger, Dhayana Dallmeier, Thomas Seufferlein

**Affiliations:** 1 Klinik für Innere Medizin 1, Universitätsklinikum Ulm, Ulm, Deutschland; 2 Zentrale Einrichtung Klinische Chemie, Universitätsklinikum Ulm, Ulm, Deutschland; 3 Institut für Virologie, Universitätsklinikum Ulm, Ulm, Deutschland; 4 Institut für Klinische Transfusionsmedizin und Immungenetik Ulm, Deutsches Rotes Kreuz Blutspendedienst Baden-Württemberg-Hessen und Universitätsklinikum Ulm, Ulm, Deutschland; 5 Kreis-Katastrophenschutz & Notfalldienst Alb-Donau Kreis, Ulm, Deutschland; 6 Institut für Geriatrische Forschung, Universitätsklinikum Ulm, Ulm, Deutschland; 7 AGAPLESION Bethesda Klinik Ulm, Ulm, Deutschland; 8 Geriatrisches Zentrum Ulm/Alb-Donau, Ulm, Deutschland; 9 Department of Epidemiology, Boston University School of Public Health, Boston, USA

**Keywords:** COVID-19, SARS-CoV‑2, Pflegeeinrichtungen, Risikopopulation, Psychische Belastung, COVID-19, SARS-CoV‑2, Care facilities, Risk populations, Psychological burden

## Abstract

**Hintergrund:**

Pflegeeinrichtungen sind Belastungen der COVID-19-Pandemie gegenüber besonders exponiert, sowohl in personellen wie strukturellen Bereichen.

**Ziel der Arbeit:**

Prospektive Querschnittsstudie zum punktuellen Infektionsgeschehen, zu psychosozialen Belastungen und zum Umgang der Einrichtungen mit der COVID-19-Pandemie.

**Material und Methoden:**

Systematische Datenerhebung zwischen dem 27.07.2020 und dem 25.08.2020 in 7 Pflegeeinrichtungen in Baden-Württemberg. Dies beinhaltete für Bewohner/Mitarbeiter einen Fragebogen, eine SARS-CoV-2-PCR und Antikörpertestung. Die Einrichtungen wurden auf Umgang und Präventionsmaßnahmen befragt.

**Ergebnisse:**

Von insgesamt 829 SARS-CoV-2-PCR-Tests waren 100 % negativ. 2 Probanden hatten SARS-CoV-2-Antikörper, allerdings ohne positive Anamnese. Keiner der Probanden mit positiver PCR in der Anamnese (*n* = 6) hatte nachweisbare Antikörper. Mitarbeiter hatten Angst, Mitmenschen, v. a. Heimbewohner, (54,4 %) anzustecken, weniger sich selbst (27,2 %). Als pandemieassoziierte Belastungen wurden in 17,1 % Erschöpfung, 16 % finanzielle Ängste und 13,1 % Schlafstörungen angegeben. Die Bewältigungsstrategien umfassten einen moderaten Anstieg schädlichen Konsumverhaltens (+3,3 % Alkohol, +4,3 % Nikotin). Wesentlich kritischer war dies bei unter 35-Jährigen (+13 % Alkohol, +12,7 % Nikotin). Frauen gaben eine Zunahme des Medikamentengebrauchs um 2,4 % an. 49,8 % der Befragten reduzierten ihre Sozialkontakte, 76,8 % veränderten ihr Hygieneverhalten. Die Einrichtungen waren eingeschränkt auf die COVID-19-Pandemie vorbereitet.

**Diskussion:**

Trotz der niedrigen Punktprävalenz zum Zeitpunkt der Erhebung belastete die COVID-19-Pandemie die Pflegeeinrichtungen in vielfachen Aspekten. Aus den entstandenen Belastungen bei Mitarbeitern müssen Bewältigungs- und Präventionskonzepte resultieren.

**Zusatzmaterial online:**

Zusätzliche Informationen sind in der Online-Version dieses Artikels (10.1007/s00391-021-01931-6) enthalten.

## Abstract (en)

**Background:**

Care facilities are particularly challenged by the COVID-19 pandemic. Besides others this includes human and structural resources.

**Objective:**

This cross-sectional study evaluated the occurrence of infections, psychosocial stress and the different strategies to handle the COVID-19 pandemic in care facilities.

**Material and methods:**

Data collection took place in 7 care facilities in Baden-Württemberg, Germany between 17 July and 25 August 2020. This included a SARS-CoV‑2 PCR and antibody testing and a questionnaire for residents and staff. Care facilities were questioned on interventions and preventive measures taken.

**Results:**

Out of 829 SARS-CoV‑2 PCR tests all remained negative. Only 2 asymptomatic subjects had detectable SARS-CoV‑2 antibodies. All subjects (*n* = 6) with a history of positive PCR had no detectable antibodies. Healthcare workers were mainly worried about infecting family, friends and especially residents (54.4%) with less fear to infect themselves (27.2%). Individual stress caused by the COVID-19 pandemic: 17.1% exhaustion, 16% financial burden and 13.1% sleeping disorders. Coping strategies included a moderate increase of harmful behavior (+3.3% alcohol, +4.3% nicotine). This was relevantly more important in staff aged under 35 years (+13% alcohol, +12.7% nicotine). Women reported a 2.4% increased use of medication, 49.8% of respondents reduced their social contacts, 76.8% changed their individual hygiene behavior. Care facilities felt prepared to a limited extent for the challenges faced by the pandemic.

**Conclusion:**

Even with a low prevalence of infections at the time of the survey the COVID-19 pandemic challenged care facilities at multiple levels. This should result in better preventive management and coping strategies.

## Hintergrund

Im Dezember 2019 wurde erstmalig das neuartige Coronavirus SARS-CoV‑2 („*s*evere *a*cute *r*espiratory *s*yndrome *co*rona*v*irus *2*“) beschrieben [17], mit ersten Infektionen im Januar 2020 in Deutschland [10]. Die Infektion mit SARS-CoV‑2 kann zur COVID-19-Erkrankung führen, mit teilweise sehr schweren Verläufen einer viralen Pneumonie [12]. Der Großteil der Infektionen verläuft asymptomatisch bis mild [12]. Als besondere Risikofaktoren gelten höheres Lebensalter, Komorbiditäten wie chronische Herzerkrankungen, Diabetes und Übergewicht [4]. Dies trifft insbesondere auf Bewohner von Pflegeeinrichtungen zu und weist diese als Hochrisikobereich aus. Neben unmittelbaren gesundheitlichen Risiken erzeugen Maßnahmen wie z. B. Kontaktbeschränkungen, Besuchsverbote und Isolationsmaßnahmen relevante Belastungen. Dies führt zu einer besonderen Verantwortung der Mitarbeiter, wobei deren erhöhte psychische Belastung und Ängste den Pandemieverlauf durch maladaptive Verhaltensweisen negativ beeinflussen können [6]. Für diesen besonders sensiblen Bereich finden sich kaum systematisch erhobene Daten [5].

Diesbezüglich wurde im Sommer 2020 prospektiv das punktuelle Infektionsgeschehen, psychosoziale Belastungen und Bewältigungsstrategien durch die COVID-19-Pandemie in ausgewählten Pflegeeinrichtungen evaluiert.

## Methode

**Figure 1:**
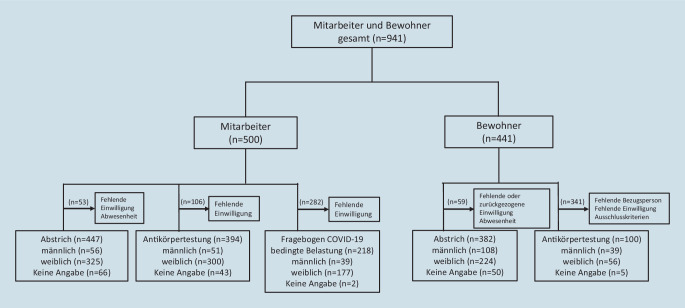

Zwischen dem 27.07.2020 und dem 25.08.2020 führte das Universitätsklinikum Ulm, im Rahmen einer in Baden-Württemberg flächendeckenden Coronareihentestung in Pflegeheimen, eine Querschnittsstudie in 7 Einrichtungen im Alb-Donau-Kreis (ADK) und in der Großstadt Ulm durch. Dies beinhaltete die Testung auf SARS-CoV‑2 im Rachenabstrich, Antikörper (AK) im Blutserum und einen strukturierten Fragebogen zu COVID-19-bedingten Belastungen unter Mitarbeitern (Zusatzmaterial online: Supplement 1). Die Studie war für alle Bewohner und Mitarbeiter offen. Vor Abstrich und Blutentnahme erfolgte eine ärztliche Aufklärung der Probanden, bei gesetzlich betreuten Personen erfolgte diese über den Betreuer. Die AK-Testung wurde nach Vorgabe der Ethikkommission nur einwilligungsfähigen Probanden angeboten. 382 Bewohnern (BW) und 447 Mitarbeiter (MA) erhielten einen Abstrich. Die AK-Testung erfolgte bei 100 BW und 394 MA; die Ergebnisse wurden den Probanden schriftlich mitgeteilt (Abb. 1). COVID-19-bedingte Belastungen wurden entsprechend einer 5‑Punkte-Likert-Skala (0: stimme überhaupt nicht zu bis 4: stimme vollständig zu) abgefragt. Weitere methodische Details finden sich im Supplement. Alle studienspezifischen Maßnahmen erfolgten durch Ärzte, unter Einhaltung geltender Hygienemaßnahmen. Eine Begutachtung und Bewilligung der Studie erfolgten durch die Ethikkommission der Universität Ulm (Nummer 186/20).

## Ergebnisse

### Basischarakteristika der Probanden

An der Studie nahmen 7 Pflegeeinrichtungen teil, 6 davon mit Spezialisierung auf die Versorgung von alten Menschen, eine auf die Unterstützung von Menschen mit Behinderung. Von 941 in den Einrichtungen gemeldeten Mitarbeitern und Bewohnern nahmen 829 (88,1 %) teil, verteilt auf 447 Mitarbeiter und 382 Bewohner. Die Rücklaufquote der Fragebögen für Basisinformationen war 84,6 % (*n* = 701, 378 MA, 323 BW). Durchschnittlich waren Mitarbeiter 46,7 und Bewohner 82,2 Jahre alt, mit einem BMI von 26,9 kg/m^2^ und 26,3 kg/m^2^. Der Frauenanteil lag bei 85,3 % der Mitarbeiter und bei 67,5 % der Bewohner. Aktive Raucher waren 30,5 % der Mitarbeiter und 4,9 % der Bewohner. Die häufigsten Komorbiditäten waren bei Mitarbeitern Arthrose (17,6 %) und arterielle Hypertonie (aHT) (14,4 %), bei Bewohnern aHT (62,4 %), demenzielle (46,8 %) und psychiatrische Erkrankungen (39,3 %) (Tab. 1). Bewohner hatten einen median FRAIL Score von 2 (0–5).

**Table 1:** 

	Mitarbeiter		Bewohner	
	*n*		*n*	
Alter, Mittelwert (min–max)	378	46,7 (17–76)	323	82,2 (27–104)
Geschlecht, *n* (%)	379	–	321	–
Frauen	–	325 (85,3)	–	224 (67,5)
Männer	–	56 (14,7)	–	108 (32,5)
BMI, Mittelwert (min–max)	376	26,9 (16,4–48,3)	313	26,3 (11,6–46,1)
Nikotin regelmäßig, *n* (%)	383	117 (30,5)	218	11 (5,1)
Alkohol regelmäßig bis gelegentlich, *n* (%)	367	101 (27,5)	225	11 (4,9)
Komorbiditäten, *n* (%)	–	–	–	–
Arterielle Hypertonie	368	53 (14,4)	295	184 (62,4)
Diabetes mellitus	366	20 (5,5)	252	80 (31,3)
Asthma	372	17 (4,6)	236	12 (5,1)
COPD	365	6 (1,6)	242	21 (8,7)
Arthrose, rheumatische Erkrankungen	369	65 (17,6)	254	84 (33,1)
Demenz	370	–	269	126 (46,8)
M. Parkinson	369	1 (0,3)	246	24 (9,8)
Psychiatrische Erkrankungen	366	16 (4,4)	257	101 (39,3)
Herzinsuffizienz	368	4 (1,1)	257	83 (32,3)
Koronare Herzerkrankung	372	6 (1,6)	248	48 (19,4)
Vorhofflimmern	365	2 (0,5)	255	52 (20,4)
Niereninsuffizienz	371	1 (0,3)	248	69 (27,8)
Leberzirrhose	370	–	234	5 (2,1)
pAVK	363	2 (0,6)	234	14 (6)
Apoplex	365	1 (0,3)	237	36 (15,2)
Osteoporose	363	12 (3,3)	254	55 (21,7)
Chronische Infektionskrankheiten	368	11 (3)	236	3 (1,3)
Hepatitis	368	7 (1,9)	235	1 (0,4)
Krebserkrankung	368	15 (4,1)	228	44 (19,3)
FRAIL Score, Median (Q1, Q3)	–	–	225	2 (0, 5)
Symptome in den 2 Wochen vor dem Abstrich, *n* (%)	386	69 (17,9)	236	8 (3,4)
Symptome seit Februar 2020, *n* (%)	373	81 (21,7)	182	13 (7,1)

### Reihenabstrichtestung auf SARS-CoV-2 in Pflegeeinrichtungen

Bei 829 Probanden (447 MA, 382 BW) erfolgte ein Rachenabstrich (Abb. 1). Alle Abstriche wurden mittels PCR negativ auf SARS-CoV‑2 getestet. Im Studienzeitraum lagen die SARS-CoV-2-Prävalenzen im Mittel bei 360,2 Fällen im ADK und 283,4 Fällen/100.000 Einwohner in Ulm.

**Figure 2:**
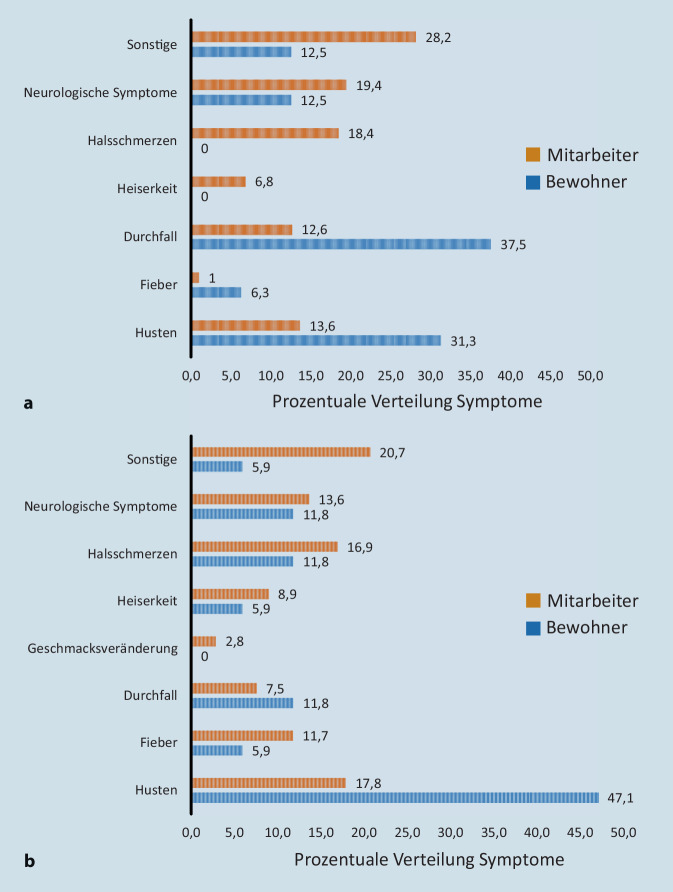

In den 2 Wochen vor dem Abstrich gaben 17,9 % der Mitarbeiter (v. a. neurologische Symptome, Halsschmerzen, Husten) und 3,4 % der Bewohner (v. a. Durchfall, Husten) COVID-19-verdächtige Symptome an (Abb. 2a; „sonstige Symptome“: Zusatzmaterial online: Supplement 2).

### Korrelation zwischen SARS-CoV-2-Antikörpern und Krankheitssymptomen

394 Mitarbeiter und 100 Bewohner wurden auf Antikörper getestet (Abb. 1). Aus dieser Gruppe hatten seit Februar 20,5 % (*n* = 79) der MA mindestens ein COVID-19-verdächtiges Symptom (v. a. Halsschmerzen, Husten) und 8 % (*n* = 8) der BW (v. a. Durchfall, Husten) (Abb. 2b; „sonstige Symptome“: Zusatzmaterial online: Supplement 2). Bei zwei Probanden (0,4 %) wurden SARS-CoV-2-AK nachgewiesen, je ein Mitarbeiter und Bewohner unterschiedlicher Einrichtungen, beide ohne COVID-19-verdächtige Vorgeschichte. Bei fünf MA und einem BW mit anamnestisch positiver SARS-CoV-2-PCR blieb der Antikörpertest allerdings negativ.

### Belastungen der Mitarbeiter in der COVID-19-Pandemie

**Figure 3:**
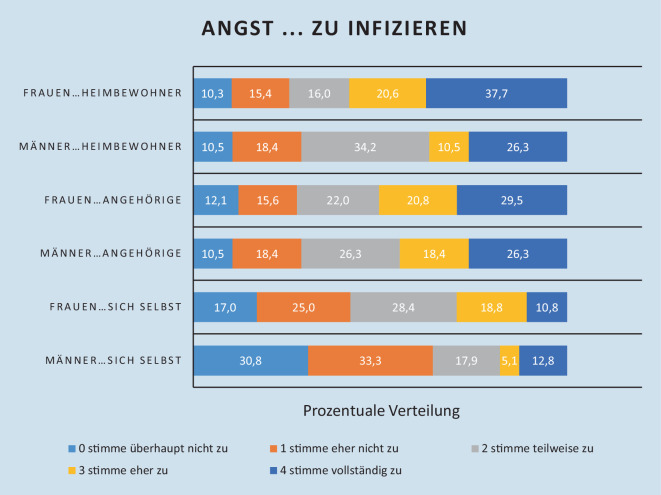

Mitarbeiter wurden gebeten, 19 Fragen der individuellen Belastung durch die Pandemie zu beantworten. 218 (57,7 %) Fragebögen konnten ausgewertet werden. Ein Mitarbeiter gab einen COVID-19-Todesfall im nahen Umfeld an, sonst gab es keine wissentlichen COVID-19-Erkrankungen oder Verstorbene im Umfeld der Befragten. Im Vordergrund stand die Sorge, Heimbewohner (54,4 %) oder Angehörige (49,3 %) und weniger sich selbst zu infizieren (27,2 %). Die 177 befragten Mitarbeiterinnen (81,2 %) gaben diese Sorgen deutlich häufiger an (Bewohner 58,3 %, Angehörige 50,3 %) als ihre 39 männlichen Kollegen (17,9 %) (BW 36,8 %, Angehörige 44,7 %). Männer hatten geringere Bedenken, sich selbst zu infizieren (64,1 % M, 42 % F) (Abb. 3).

**Figure 4:**
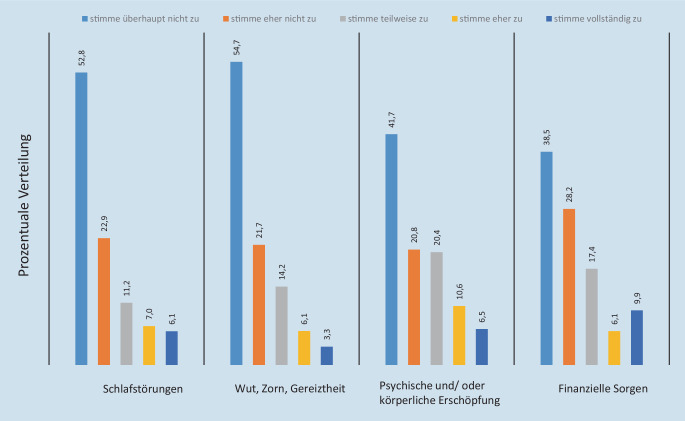

Ein relevanter Anteil der befragten Mitarbeiter gab individuelle Belastungen durch die Pandemie an. Bei 13,1 % (*n* = 28) traten vermehrt Schlafstörungen, bei 17,1 % (*n* = 37) zunehmende psychische und/oder körperliche Erschöpfung und bei 16 % (*n* = 34) finanzielle Zukunftssorgen auf. Eher stimmten der Zunahme der Belastungen 11,2 % (*n* = 24) bei Schlafstörungen, 20,4 % (*n* = 44) bei psychischer und/oder körperlicher Erschöpfung und 17,4 % (*n* = 37) bei finanziellen Sorgen zu (Abb. 4).

Vor allem bei Mitarbeiterinnen waren Erschöpfung (*n* = 32, 18,3 %) und finanzielle Unsicherheit (*n* = 32, 18,6 %) präsent, ohne signifikanten Altersunterschied. Zunehmende Aggression im Sinne von Wut, Zorn, Gereiztheit gaben 9,4 % an (Abb. 4) (7,7 % M, 9,9 % F).

**Figure 5:**
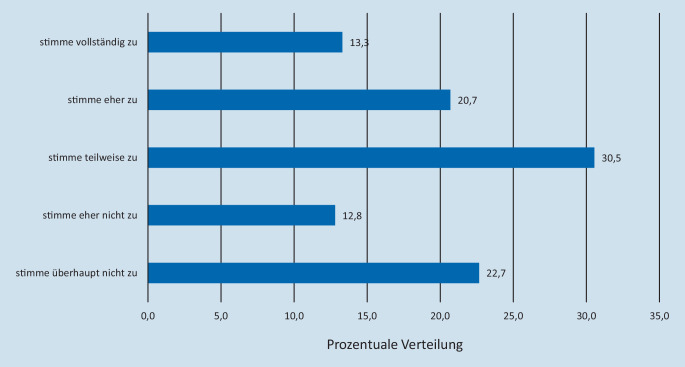

Die infolge der Pandemie getroffenen Maßnahmen führten bei 34 % der Mitarbeiter alters- und geschlechtsunabhängig zu unmittelbaren Zusatzbelastungen in der täglichen Arbeit (Abb. 5).

### Bewältigungsstrategien in der COVID-19-Pandemie

Im Rahmen der COVID-19-Pandemie gaben 3,3 % (*n* = 7) der Mitarbeiter an, mehr Alkohol als vorher zu konsumieren. Dies war geschlechtsunabhängig, jedoch bei ≤ 35-Jährigen signifikant häufiger (*n* = 6, 13 %) als bei ihren älteren Kollegen (*n* = 1, 0,6 %). Äquivalent rauchten auch 4,3 % (*n* = 9) der 117 Raucher mehr (7,7 % M, 3,5 % F). Auch hier bestand ein signifikanter Altersunterschied mit +12,7 % bei ≤ 35-Jährigen und nur +1,9 % bei Älteren. Der Gebrauch von Antidepressiva, Beruhigungsmitteln oder Schlafmitteln wurde nur von Frauen angegeben und zeigte eine geringfügige Veränderung (+2,4 %) (Tab. 2). Deutlich änderte sich das Sozialverhalten, 49,8 % (*n* = 106) reduzierten den physischen Kontakt zu Mitmenschen. Häufiger war dies bei > 35-Jährigen (52,2 %) als bei Jüngeren (41,2 %). Deutlich waren die Effekte auf das Hygieneverhalten, welches 76,8 % (*n* = 165) änderten.

**Table 2:** 

Seit dem Ausbruch der COVID-19-Pandemie...	Altersgruppe, Mitarbeiter	Stimme vollständig zu	Stimme eher zu	Stimme teilweise zu	Stimme eher nicht zu	Stimme überhaupt nicht zu
		*n* (%)	*n* (%)	*n* (%)	*n* (%)	*n* (%)
... trinke ich mehr Alkohol	≤ 35 Jahre (*n* = 46)	3 (6,5)	3 (6,5)	1 (2,2)	1 (2,2)	38 (82,6)
	> 35 Jahre (*n* = 161)	–	1 (0,6)	3 (1,9)	14 (8,7)	143 (88,8)
... rauche ich mehr	≤ 35 Jahre (*n* = 47)	5 (10,6)	1 (2,1)	1 (2,1)	4 (8,5)	36 (76,6)
	> 35 Jahr (*n* = 160)	3 (1,9)	–	5 (3,1)	12 (7,5)	140 (87,5)
... nehme ich mehr Antidepressiva/Beruhigungsmittel/Schlafmittel	≤ 35 Jahre (*n* = 46)	1 (2,2)	–	1 (2,2)	1 (2,2)	43 (93,5)
	> 35 Jahre (*n* = 161)	2 (1,2)	2 (1,2)	2 (1,2)	3 (1,9)	152 (94,4)

### Pflegeeinrichtungen in der COVID-19-Pandemie

Die Pflegeeinrichtungen sollten einschätzen, inwiefern sie sich auf die epidemische Lage im Frühjahr 2020 vorbereitet sahen (Rücklaufquote 71,4 %, *n* = 5) und gaben im Mittelwert 4,6 auf einer 10-teiligen Skala an (0: nicht- bis 10: optimal vorbereitet). Relevante Probleme in der Beschaffung adäquater Schutzausrüstung und Hygienematerialien gaben 4 von 5 Einrichtungen (80 %) anfänglich an.

Detaillierte Konzepte für Mitarbeiter orientierten sich v. a. an lokalen Voraussetzungen und beinhalteten u. a. (i) feste Teameinteilung in Wohnbereiche, mit (ii) Trennung der Umkleiden, (iii) asynchrone Übergabezeiten, (iv) Wohngruppentrennung der BW, (v) Schließung von Gemeinschaftsräumen, (vi) Verzicht auf Gemeinschaftsaktivitäten, (vii) Arbeiten mit Schutzausrüstung, (viii) regelmäßige Hygieneschulungen, (ix) verkürzte Reinigungsintervalle, (x) Vorhalten von Quarantänebereichen, (xi) Symptomscreening und (xii) bauliche Maßnahmen.

Mit Hinblick auf den Winter 2020/2021 sahen sich die Pflegeeinrichtungen ausreichend vorbereitet und bewerteten den Stand ihrer Vorbereitungen im Mittel mit 8,6 von 10 Punkten.

### Akzeptanz der COVID-19-Pandemie bedingten Maßnahmen

Der Großteil der Mitarbeiter (63,4 %) fühlte sich ausreichend durch die behördlich angeordneten und im Heim durchgeführten Maßnahmen geschützt. Weiterhin empfanden 10 % der Befragten die getroffenen Maßnahmen als unzureichend. Die in den Einrichtungen umgesetzten Maßnahmen, insbesondere Isolationsmaßnahmen, wurden von 78,5 % unterstützt. Die Kommunikation in den Pflegeeinrichtungen wurde von 70,5 % der Mitarbeiter positiv bewertet, Tab. 3.

**Table 3:** 

Seit dem Ausbruch der COVID-19-Pandemie...	Altersgruppe, Mitarbeiter	Stimme vollständig zu	Stimme eher zu	Stimme teilweise zu	Stimme eher nicht zu	Stimme überhaupt nicht zu
		*n* (%)	*n* (%)	*n* (%)	*n* (%)	*n* (%)
... fühle ich mich durch die Maßnahmen des Heimes und der Behörden geschützt	≤ 35 Jahre (*n* = 50)	12 (24)	16 (32)	17 (34)	4 (8)	1 (2)
	> 35 Jahre (*n* = 160)	47 (29,4)	58 (36,3)	39 (24,4)	8 (5)	8 (5)
... fühle ich mich immer gut informiert durch die Heimleitung	≤ 35 Jahre (*n* = 50)	16 (32)	17 (34)	12 (24)	3 (6)	2 (4)
	> 35 Jahr (*n* = 161)	64 (39,8)	52 (32,3)	22 (13,7)	9 (5,6)	14 (8,7)
... empfinde ich die Isolierungsmaßnahmen in unserem Pflegeheim übertrieben	≤ 35 Jahre (*n* = 51)	2 (3,9)	1 (2)	7 (13,7)	16 (31,4)	25 (49)
	> 35 Jahre (*n* = 160)	7 (4,4)	7 (4,4)	22 (13,8)	22 (13,8)	102 (63,8)
... habe ich die Pandemie als Realität anerkannt und akzeptiert	≤ 35 Jahre (*n* = 49)	26 (54,2)	12 (25)	4 (8,3)	4 (8,3)	2 (4,2)
	> 35 Jahre (*n* = 162)	88 (54,3)	42 (25,9)	21 (13)	3 (1,9)	8 (4,9)
... habe ich meine Einstellung dazu verändert, was mir im Leben wirklich wichtig ist	≤ 35 Jahre (*n* = 50)	9 (18)	9 (18)	12 (24)	6 (12)	14 (28)
	> 35 Jahre (*n* = 159)	25 (15,7)	22 (13,8)	48 (30,2)	31 (19,5)	33 (20,8)

Die Akzeptanz der COVID-19-Pandemie als Teil der eigenen Realität lag bei 79,8 % (*n* = 170). Immerhin 17 Mitarbeiter (8 %) verneinten die Pandemie, Männer (12,8 %) häufiger als Frauen (7 %) (Tab. 3). Einen unmittelbaren Einfluss der Pandemie auf die eigene Lebenseinstellung gaben 30,6 % (*n* = 65) an.

## Diskussion

Ergänzend zu einer Reihenabstrichtestung in Pflegeeinrichtungen führten wir eine Befragung mit guter Teilnahmequote (86,6 % BW, 89,4 % MA) durch. Die COVID-19-Prävalenz war in den Einrichtungen zwar niedrig, Mehrbelastung von Mitarbeitern auf psychischer und physischer Ebene sowie ein kritisch zu bewertender Umgang damit jedoch häufig. Gründe der Nichtteilnahme beruhten v. a. auf räumlicher Abwesenheit aufgrund von Urlaub, einer längeren dienstfreien Zeit oder Krankheit. Die zwingend vorgegebene Einwilligungsfähigkeit für die Antikörpertestung schloss einen größeren Anteil der Bewohner aus, folglich nahmen 78,8 % der Mitarbeiter und 22,7 % der Bewohner teil.

Die Reihentestung in Baden-Württemberg wurde aufgrund der zu diesem Zeitpunkt noch unklaren Prävalenz asymptomatischer SARS-CoV-2-Infektionen in Pflegeeinrichtungen durchgeführt. Bei 100 % negativen SARS-CoV-2-PCR, selbst bei unmittelbar vorher symptomatischen Probanden, muss dieses Konzept in Abwesenheit eines Indexfalls und einer niedrigen Gesamtinzidenz in der Bevölkerung, kritisch hinterfragt werden. Ein hoher Ressourcenaufwand zur Testung steht einer niedrigen Vortestwahrscheinlichkeit gegenüber. Ob Reihentestungen mit Antigenschnelltesten diese Lücke sicher schließen können, bleibt fraglich, da diese eine gewisse Antigenlast voraussetzen, die nicht bei allen Infizierten vorliegt und somit bei negativem Antigentest trotzdem Kontagiösität bestehen kann [3] sowie die Sensitivität und Spezifizität bei Virusvarianten noch unklar sind [11]. Aktuell sind Antigentestungen in Pflegeeinrichtungen jedoch vorgegeben und erscheinen als Screeningmethode nach derzeitigem Kenntnisstand in Ergänzung zu spezifischen Hygienemaßnahmen und AHA + L-Regeln sinnvoll [11].

Auffallend ist, dass bei allen 6 Teilnehmern mit einer positiven PCR in der Anamnese der Antikörpertest negativ war. Gleichzeitig wurden Antikörper bei 2 Teilnehmern festgestellt, welche keine positive Anamnese oder keinen positiven PCR-Test bisher hatten. Hier könnte die zeitliche Latenz zwischen Infektion und Testung eine Rolle spielen [2]. Entsprechend einer Cochrane-Analyse ist der Nachweis 21 bis 35 Tage nach der Infektion am akkuratesten [2]. Ein teilweise schneller Abfall von SARS-CoV-2-AK findet sich v. a. bei milden COVID-19-Verläufen [13], entsprechend dem untersuchten Kollektiv. Eigene Ergebnisse aus dem Universitätsklinikum Ulm an über 650 Probanden zeigten eine falsch-negativ Rate von etwa 3 % der Antikörperteste, während falsch-positive nicht beobachtet wurden (unveröffentlichte Daten).

Die teilnehmenden Pflegeeinrichtungen kamen gut durch die erste Pandemiephase im Frühjahr, trotz zunächst eingeschränkter Ressourcen. Als Überbrückungsmaßnahmen wurden z. B. Einwegmaterialien mehrfach verwendet und Mund-Nasen-Schutzmasken anstelle von FFP2-Masken. Dies beruht mitunter auf der schnellen Umsetzung spezifischer Maßnahmen in den Einrichtungen, trotz einer verglichen mit Gesamtdeutschland höheren SARS-CoV-2-Inzidenz [7, 9]. Versorgungslücken wurden im Laufe des Frühjahres durch das Land und/oder zentrale Großbeschaffungen in den Unternehmensgruppen geschlossen. Trotz der zunächst unklaren Entwicklung der Pandemie erließ keine der Einrichtungen einen Aufnahmestopp für neue Bewohner. Mitte September waren die Einrichtungen zuversichtlich gegenüber ansteigenden Infektionszahlen. Die Mitarbeiter zeigten ein deutliches Vertrauen in staatliche Institutionen, die Heimleitungen und die getroffenen Maßnahmen. Dies deckt sich mit Erhebungen im Bevölkerungsquerschnitt Erwachsener in Deutschland [8].

Die Pandemie führt jedoch in der täglichen Arbeit mit den Bewohnern zu einer deutlichen Mehrbelastung der Mitarbeiter und resultiert in vermehrter körperlicher und psychischer Erschöpfung, Schlafstörungen und Aggressionen. Die daraus folgende Zunahme schädlichen Konsumverhalten ist besonders bei jungen Menschen kritisch herauszustellen. Unsere Daten stehen teilweise entgegen der Gesamtbevölkerung, welche durchschnittlich weniger Alkohol konsumierte [14]. Andere Arbeiten berichten eine moderate Zunahme des Alkoholkonsums, insbesondere für Personengruppen, die in der Pandemie unter erhöhter Belastungen standen [16]. Eine systematische Erhebung für Mitarbeiter in Gesundheitsberufen ähnlich dieser findet sich in der publizierten Literatur bisher nicht. Besonders nachhaltig waren die Effekte auf das „social distancing“ und ein geändertes Hygieneverhalten. Dies ist mitunter Folge von Öffentlichkeitskampagnen [18], Schulungen und Zugangsbeschränkungen in den Einrichtungen [15].

Als limitierend in dieser Studie müssen die fehlende Erfassung von individuellen Belastungen der Heimbewohner, ein möglicherweise bestehender Selektionsbias hin zu eher weniger kritischen Mitarbeitenden, eine reduzierte Anzahl an AK-Testungen bedingt durch die Einschlusskriterien bei BW und Verzerrungseffekte durch die Beschränkung auf 7 Einrichtungen genannt werden.

Zusammenfassend zeigt die hier vorliegende Studie in Pflegeeinrichtungen eines Kreises und einer Großstadt in Baden-Württemberg eine relevante Mehrbelastung der Mitarbeiter, diskrepant zum tatsächlichen Ausmaß des Infektionsgeschehens. Es wurden relevante Beeinträchtigungen der täglichen Arbeit durch die im Rahmen der Pandemie getroffenen Maßnahmen und eine größere Sorge vor Ansteckung von Heimbewohnern und Angehörigen angegeben. Relevant scheint auch ein leicht erhöhter Suchtmittelkonsum, vor dem Hintergrund des in diesem Bereich üblicherweise bestehenden negativen Berichtsbias [1]. Die Konzepte zur Bewältigung der Pandemie wurden von den Mitarbeitern akzeptiert, müssen aber weiter verbessert werden. Kurzfristig sollten aber vor allen Dingen Konzepte umgesetzt werden, die die Mehrbelastung der Mitarbeiter auffangen.

## Fazit für die Praxis

- Die COVID-19-Pandemie führt zu relevanten Belastungen von Mitarbeitern in Pflegeeinrichtungen, mit teilweise kritischen Bewältigungsstrategien trotz zum Zeitpunkt der Untersuchung niedrigem Infektionsgeschehen.
- Kurzfristig müssen Konzepte zur Bewältigung der relevanten Mehrbelastung der Mitarbeiter angeboten werden.

## Supplementary Information





