# Supplementary material for: COVID-19 pandemic-related burden and SARS-CoV-2 prevalence in care facilities
Source: Z Gerontol Geriatr. 2021 Jul 14;54(5):463–70. [Article in German] doi: 10.1007/s00391-021-01931-6 (PMC8278814; doi:10.1007/s00391-021-01931-6)
Supplement: Supplementary file 1 [file 391_2021_1931_MOESM1_ESM.pdf]

Supplement

Supplement 1. Fragebogen Mitarbeiter

**Seit dem Ausbruch der Covid-19-Pandemie …**	**Stimme überhaupt nicht zu** (0)	**Stimme eher nicht zu** (1)	**Stimme teils, teils zu** (2)	**Stimme eher zu** (3)	**Stimme vollständig zu** (4)
1. …habe ich Angst, mich zu infizieren.	☐	☐	☐	☐	☐
2. …habe ich Angst, Angehörige oder die Familie zu infizieren.	☐	☐	☐	☐	☐
3. …habe ich Angst Heimbewohner zu infizieren.	☐	☐	☐	☐	☐
4. …fühle ich mich durch die Maßnahmen des Heimes und der Behörden ausreichend geschützt.	☐	☐	☐	☐	☐
5. …habe ich mein persönliches Hygieneverhalten verändert	☐	☐	☐	☐	☐
6. …leide ich vermehrt unter Schlafproblemen.	☐	☐	☐	☐	☐
7. …fühle bzw. verhalte ich mich deutlich gereizter, wütender, zorniger	☐	☐	☐	☐	☐
8. …fühle ich mich vermehrt körperlich oder psychisch erschöpft.	☐	☐	☐	☐	☐
9. …habe ich jeglichen körperlichen Kontakt zu Mitmenschen reduziert	☐	☐	☐	☐	☐
10. … mache ich mir vermehrt Sorgen um die eigene finanzielle Situation	☐	☐	☐	☐	☐
11. …habe meine Einstellung dazu verändert, was mir im Leben wirklich wichtig ist.	☐	☐	☐	☐	☐
12. …rauche ich mehr.	☐	☐	☐	☐	☐
13. …trinke ich mehr Alkohol.	☐	☐	☐	☐	☐
14. …nehme ich mehr Antidepressiva/ Beruhigungsmittel/Schlafmittel.	☐	☐	☐	☐	☐
15. …empfinde ich die Isolierungs-Maßnahmen in unserem Pflegeheim übertrieben	☐	☐	☐	☐	☐
16. …empfinde ich die Situation besonders belastend für die Heimbewohner	☐	☐	☐	☐	☐
17. …beinträchtigen die getroffenen Maßnahmen meine Arbeit mit den Heimbewohnern	☐	☐	☐	☐	☐
18. …fühle ich mich immer gut informiert durch die Heimleitung	☐	☐	☐	☐	☐
19. …habe ich die Pandemie als Realität anerkannt und akzeptiert	☐	☐	☐	☐	☐

Supplement 2: Sonstige Symptome

Freitextangaben der Probanden zu Sonstigen Symptomen in den letzten 2 Wochen vor Studienteilnahme

	Bewohner	Mitarbeiter
Müdigkeit	2	24
Gliederschmerzen	0	10
Abgeschlagenheit	0	13
Appetitlosigkeit	1	0

Freitextangaben der Probanden zu Sonstigen Symptomen seit Februar 2020

	Bewohner	Mitarbeiter
Müdigkeit	0	29
Gliederschmerzen	1	16
Abgeschlagenheit	0	14
Nasenbluten	0	1
Ohrenschmerzen	0	2
Appetitlosigkeit	0	1
Harnwegsinfekt	1	0
Schnupfen	0	1
Thrombose	0	1
Schwächeanfall	0	1
