# Supplementary material for: COVID-19 pandemic-related burden and SARS-CoV-2 prevalence in care facilities
Source: Z Gerontol Geriatr. 2021 Jul 14;54(5):463–70. [Article in German] doi: 10.1007/s00391-021-01931-6 (PMC8278814; doi:10.1007/s00391-021-01931-6)
Supplement: Supplementary file 2 [file 391_2021_1931_MOESM2_ESM.docx]

**Online Supplement Methoden**

**Abstrich auf SARS-CoV-2**

Die Abstriche wurden einheitlich bei allen Probanden aus dem Rachen entnommen mit einem Abstrich System (BD MAX Specimen Collection Kit) der Firma BD (Becton, Dickinson and Company) ohne Trägerlösung. Die Abstrichtupfer wurden für 5 min unter ständiger Agitation in 2 ml Pufferlösung (cobas PCR media Kit) inkubiert. Die Inkubation erfolgte auf einem Schüttler Vibrax-VXR bei Mot 100/min. Der Nachweis von SARS-CoV-2 spezifischer Nukleinsäure erfolgte durch RT-PCR unter Verwendung des Cobas SARS-CoV-2 Kits und eines Cobas 6800 Systems der Firma Roche.

**Antikörpertestung auf SARS-CoV-2**

Zur Antikörper Testung wurde das Roche System Cobas e 801 sowie der zugehörige Assay Elecsys Anti-SARS-CoV-2 von Roche verwendet. Als cut-off Werte der Messung wurden die vom Hersteller Roche vorgegebenen Angaben verwendet. Die Testung erfolgte aus 7,5ml Serum (SARSTEDT S-Monovette Serum-Gel, 7,5ml) über die Zentrale Einrichtung Klinische Chemie am Universitätsklinikum Ulm. Die Ergebnisse der Antikörpertestung wurden den Probanden schriftlich zur Verfügung gestellt.

**Strukturierter Fragebogen für Mitarbeiter und Bewohner von Pflegeeinrichtungen**

Es wurden Mitarbeiter- und Bewohnerfragebögen erstellt, mit unterschiedlichen Items, welche epidemiologische Basisdaten, Vorerkrankungen, Medikation, COVID19-assoziierte Symptome und psychosoziale Aspekte einbezogen haben (Tabelle 1 und Supplement 1). Die Auswertung erfolgte anonymisiert. Der psychosoziale Fragebogenteil wurde nur einwilligungsfähigen Probanden angeboten. Psychosoziale Aspekte wurden mit insgesamt 19 Einzelfragen bei Mitarbeitern abgefragt. Die COVID19-bedingten Belastungen konnten auf einer 5 Punkte Likert-Skala angegeben werden: (0) Stimme überhaupt nicht zu, (1) Stimme eher zu, (2) Stimme teils, teils zu, (3) Stimme eher zu, (4) Stimme vollständig zu.

**Covariablen**

Teilnehmer wurden mittels Fragebogen nach soziodemographischen Daten inkl. Alter, Geschlecht, Größe, Gewicht gefragt. Es wurde Information über Rauchen (aktiver Raucher, Nichtraucher seit wann, Nieraucher), Alkoholkonsum (aktiver Konsum mit Menge, Abstinenz) erhoben. Komorbiditäten wurden analog Charlson Comorbidity Index abgefragt. Die Gebrechlichkeit wurde mittels FRAIL Score erhoben.

**Statistische Analyse**

Die Datenauswertung erfolgte mittels deskriptiver Statistik, es wurden i.d.R. Mittelwerte, Absolutwerte oder prozentuale Verhältnisse zum definierten Kollektiv angegeben. Mittels Chi^2^-Test wurden Häufigkeiten zwischen Gruppen verglichen, als signifikant wurde p<0,05 angenommen. In der Auswertung der Mitarbeiterfragebögen wurden die Kategorien „Stimme vollständig zu“ und „Stimme eher zu“, sowie „Stimme überhaupt nicht zu“ und „Stimme eher nicht zu“ zusammengefasst. Zur Datenauswertungen und Abbildungserstellung wurde Microsoft Excel 2013 verwendet.
